# 27 Streamlining the Discharge Process

**DOI:** 10.1093/jbcr/irae036.027

**Published:** 2024-04-17

**Authors:** Rebecca Coffey, Elizabeth Salazar, Amber Ewing, Janie J Faris, Alina Ruiz, Michelle Terry

**Affiliations:** Parkland Health, Keller, TX; Parkland Health, Garland, Texas; Parkland Health, Dallas, Texas; Parkland Health, Frisco, Texas; Parkland Health, Keller, TX; Parkland Health, Garland, Texas; Parkland Health, Dallas, Texas; Parkland Health, Frisco, Texas; Parkland Health, Keller, TX; Parkland Health, Garland, Texas; Parkland Health, Dallas, Texas; Parkland Health, Frisco, Texas; Parkland Health, Keller, TX; Parkland Health, Garland, Texas; Parkland Health, Dallas, Texas; Parkland Health, Frisco, Texas; Parkland Health, Keller, TX; Parkland Health, Garland, Texas; Parkland Health, Dallas, Texas; Parkland Health, Frisco, Texas; Parkland Health, Keller, TX; Parkland Health, Garland, Texas; Parkland Health, Dallas, Texas; Parkland Health, Frisco, Texas

## Abstract

**Introduction:**

Survival rates of burn patients are increasing but the challenges for hospital discharge are numerous and require a comprehensive approach for a successful discharge. The unit-based council of a regional burn center identified two reasons for readmissions; inadequate education and preparation prior to discharge. The purpose of this quality project was to evaluate the discharge processes, identify opportunities and improve patient outcomes.

**Methods:**

Data was collected on readmission rates and discharge instruction accuracy prior to implantation of the new discharge process (NDP). After development of the NDP education was provided to all burn team members. All discharged patients during the evaluation period from 6/1 to 8/31/2023 were reviewed. Data collection included: readmission rates, number of patients receiving discharge phone calls (9/1/2023 – 9/28/202), and the use of the new discharge instructions.

**Results:**

A total of 478 discharges occurred during the evaluation period. New discharge instructions were used 38.5% versus old instructions 61.5%. Twenty-eight patients (100%) received discharge phone calls; all patients understood wound care instructions. One patient had difficulty completing wound care, 2 patients did not receive supplies, and 3 patients did not pick up the prescription medications. Readmission rates improved to 7% with, wound care (30.7%), cellulitis (30.7%) and uncontrolled pain (7%) as the most common reasons for readmission. All burn patients received the new discharge education folders with references to the newly completed wound care videos.

**Conclusions:**

A systematic multidisciplinary approach to the discharge process will provide safe and effective discharge for the burn patient. Education of all team members is imperative for successful change.

**Applicability of Research to Practice:**

Identifying gaps in the discharge process will improve long term outcomes for patients.

